# Causality, Machine Learning, and Feature Selection: A Survey

**DOI:** 10.3390/s25082373

**Published:** 2025-04-09

**Authors:** Asmae Lamsaf, Rui Carrilho, João C. Neves, Hugo Proença

**Affiliations:** 1 IT: Instituto de Telecomunicações, University of Beira Interior, 6200-001 Covilhã, Portugal; rui.carrilho@ubi.pt (R.C.); hugomcp@di.ubi.pt (H.P.); 2 Department of Computer Science, University of Beira Interior, 6200-209 Covilhã, Portugal; jcneves@di.ubi.pt

**Keywords:** causality, causal inference, causal discovery, machine learning, feature selection, feature extraction, sensor data

## Abstract

Causality, which involves distinguishing between cause and effect, is essential for understanding complex relationships in data. This paper provides a review of causality in two key areas: causal discovery and causal inference. Causal discovery transforms data into graphical structures that illustrate how variables influence one another, while causal inference quantifies the impact of these variables on a target outcome. The models are more robust and accurate with the integration of causal reasoning into machine learning, improving applications like prediction and classification. We present various methods used in detecting causal relationships and how these can be applied in selecting or extracting relevant features, particularly from sensor datasets. When causality is used in feature selection, it supports applications like fault detection, anomaly detection, and predictive maintenance applications critical to the maintenance of complex systems. Traditional correlation-based methods of feature selection often overlook significant causal links, leading to incomplete insights. Our research highlights how integrating causality can be integrated and lead to stronger, deeper feature selection and ultimately enable better decision making in machine learning tasks.

## 1. Introduction

Causality refers to the relationship between cause and effect, where one variable or action (the cause) brings about another variable or outcome (the effect). It is the fundamental principle of understanding how things happen in the world. Causality suggests that there is a direct relationship between the occurrence of one variable and the resulting occurrence of another variable. Because of regular exercise, the body weight changed. Due to the higher temperatures, the ice melted. When we learn causality in data science, we should understand the distinction between causality and correlation. In causality, a change in one variable causes a change in another variable. The correlation implies that when one variable changes, the other variable also changes, but it does not necessarily imply a cause-and-effect relationship.

The exploration of causal relationships involves two fundamental tasks: causal discovery and causal inference. Causal discovery aims to extract the directed acyclic graph (DAG) representation of information, often sourced from tabular data. The initial phase of this process involves identifying the correlations between variables, followed by determining the direction of causation in a subsequent step or finding hidden factors(unobserved variables) that affect the variables you are studying (observed variables). Causal inference, on the other hand, seeks to quantitatively estimate the associations between variables. This task may begin with a complete or an incomplete causal structure. In scenarios with incomplete structure, a specialized causal inference tool can identify absent connections or confounding effects. Subsequently, the process involves estimating variable values given the observations of their causes, extending to more complicated situations such as interventional or counterfactual situations.

Machine learning techniques provide a way to better estimate causal effects. In machine learning, tasks can be predictive or descriptive. However, understanding cause and effect, where we imagine changing certain variables and observing the effects on the data-generation process, is also crucial. We often ask questions like, “What would happen to certain variables, such as features or labels, if we changed one specific variable?” and “Which variables can we change to impact the value of another variable?” These types of inquiry are classified as causal inference and causal discovery, respectively.

In the field of data science and machine learning, selecting relevant features is important for making models and making predictions. Between different methods of selecting features, the use of causality for feature selection is a powerful technique. This method moves beyond normal strategies that depend on correlations, instead focusing on the cause link between features and results. However, in the sensor dataset, feature selection presents unique challenges between high dimensionality, noise data, and sensor measurements [1]. These issues resulted in spurious correlations, which makes it difficult to truly identify relevant features. Understanding the reason for such a dataset requires a careful approach to identifying and addressing the more intensive exploration of data and a careful method to identifying and addressing potential confounders, ensuring that the selected characteristics actually contribute to the model performance.

Causal-based feature selection in machine learning struggles with several issues. Firstly, it is naturally complicated to accurately identify the causal relationship between features, especially with high-dimensional data. This process can be computationally expensive and prone to errors if the underlying causal assumptions are incorrect. The sensor dataset, in particular, faces unique challenges due to its high dimensionality, noise, and interdependence among sensor measurements, which often gives rise to spurious correlations and makes the search for causal relationships more difficult. In addition, such methods often require detailed domain knowledge and a strong dataset, which may not always be available. Balancing the need for accurate causes with the availability of data and practical computational resources makes causal-based feature selection a challenging but interesting aspect of advanced analytics. By focusing on the causal links, the purpose of this method is not only to enhance the prediction power of the model, but also to improve its interpretation and reliability, especially in scenarios where the underlying causal mechanism is important to understand. In fields such as healthcare, economics, remote sensing, social sciences, and sensor-based applications, where causal relationships are fundamental, this approach promises to highlight deep insight and promote the processes of strong decisions.

In our paper, the second section is dedicated to initiating the basics of causality-its definitions, the framework of structural causal models, and the concept of potential outcomes. The third section examines the principle of causality theory, which is divided into causal inference and causal discovery. This section also reviews previous approaches for addressing causality-related issues. Subsequently, we explore how causality intersects with machine learning models, highlighting models from the existing literature and discussing their relevance. Furthermore, we analyze causal-based feature selection methods, particularly in the context of sensor applications. The conclusion of the paper identifies several unresolved challenges in the field.

## 2. Preliminaries

This section explores two critical frameworks that form the key of our understanding of causality in both statistics and machine learning: structural causal models (SCM) and the potential outcome framework. These concepts are essential for understanding and applying causality in various fields, including machine learning.

### 2.1. Definitions

This section examines two important frameworks that form the key to our understanding of both statistics and machine learning: structural causal models (SCMs) and the potential outcome. These concepts are required to understand and apply causality in various fields, including machine learning. We introduce the notation employed in the framework of causality, especially focusing on the potential outcome and causal structure models. These models provide a basis for understanding and analyzing the cause. The potential outcome framework exposes the concept of causality as it is related to the treatment or intervention applied to units and assesses the effects of these treatments by comparing potential outcomes. On the other hand, the cause structure model framework uses a more graphical approach to represent causal relationships, often incorporating nodes and edges to present the features and their interactions. We will begin with three fundamental concepts that are important for causal inference: unit, treatment, and outcome.

**A unit** [2] is the basic element of analysis in studies of treatment effects. It can be an individual, an entity, or any observational unit in a dataset or a study. Units can be a range of entities that are observed, such as people, animals, households, regions, and others, depending on the research question.

**Treatment** is the variable, action, or intervention whose causal effect on an outcome is the focus of the study.

Units are categorized into two groups: the treated group and the control group. The treated group includes units subjected to the treatment or intervention under investigation, while the control group comprises units that do not receive treatment.

**The potential outcome** refers to the outcome of the units that receive the treatment, essentially, the outcome of the treated group.

**The observed or factual outcome** is the outcome that occurs when a specific treatment, action, or intervention is applied. It is the real outcome observed in a group of units following a specific treatment or intervention.

**The counterfactual outcome** is the result of a unit having received a different treatment. It imagines the outcome for the same group of units had they not received the actual treatment or intervention under study.

Counterfactual outcomes are hypothetical results from inexperienced treatments, with only one observed potential outcome per unit and all others unobserved. In studies using observational data, where treatments and outcomes are observed, units are often collected. This information can be divided into two categories: pre-treatment variables, which are characteristics present before the treatment, and post-treatment variables, which are data collected after the treatment or intervention.

**Pre-treatment variables**, which remain unaffected by treatment and are often described as background variables, include the characteristics of units prior to treatment.

**Post-treatment variables** are those that are influenced by treatment.

**Confounder variables** are variables that influence both treatment and outcome, potentially biasing the treatment effect. These factors affect both the treatment and the outcome, which can bias the results.

The treatment effect describes the impact or influence that a specific treatment, intervention, or exposure has on an outcome variable. This effect can be observed at different levels, such as in the general population, among those who received the treatment, within particular subgroups, and at the level of individual units. To better understand this, we start by explaining the treatment effect in situations where the treatment is binary.

This explanation can be extended to include scenarios that involve multiple treatments by examining the potential outcomes of each treatment. The treatment effect is often quantified by the Average Treatment Effect (ATE), which assesses the difference in the outcomes between the units receiving treatment *t* and those not.

This concept is mathematically formulated as follows:

(1) ATE=E[Y(t=1)]−E[Y(t=0)]

Y(t=1) shows the possible consequence for the treated group, and Y(t=0) shows the possible consequence for the control group in the whole population. The Average Treatment Effect on the Treated (ATT) is the treatment effect concerning the individuals who really received the treatment, and the ATT is stated as

(2) ATT=E[Y(t=1)∣t=1]−E[Y(t=0)∣t=1]

In this case, Y(t=1)∣t=1 and Y(t=0)∣t=1 represent the potential outcomes for the treated group under treatment and control conditions.

For specific subgroups, the treatment effect is called the Conditional Average Treatment Effect (CATE), given by

(3) CATE=E[Y(t=1)∣X=x]−E[Y(t=0)∣X=x]

where Y(t=1)∣X=x and Y(t=0)∣X=x are the potential outcomes of the treated and control conditions in subgroup *x*.

The treatment effect for an individual is called the Individual Treatment Effect (ITE), and for unit *i*, is given by

(4) $${\mathrm{ITE}}_{i}=Y_{i}\left(t=1\right)-Y_{i}\left(t=0\right)$$

where $Y_{i}\left(t=1\right)$ and $Y_{i}\left(t=0\right)$ are the potential outcomes of unit *i* under treatment and control conditions. In some cases, ITE is treated as the same as CATE.

In causal inference, the main goal is to determine the effect of treatments or actions using existing data, which often requires making certain assumptions.

- **The Stable Unit Treatment Value Assumption (SUTVA)** states that in any research, individuals or units do not affect or interfere with one another. This means that the possible outcome for any person does not change based on the treatment others receive. Moreover, SUTVA holds that the expected outcomes for each person are stable, regardless of what treatments are given to others. It also suggests that there are not multiple versions or kinds of treatment that could cause varied potential outcomes for any individual.
- **Positifity** refers to the assumption that in any subset of the population defined by certain covariates or characteristics, there is a non-zero probability of receiving any level of treatment. It is a critical assumption when conducting causal inference using observational or experimental data.
- **Ignorability:** In the case of non-randomized studies, the treatment is assumed to be independent if (after controlling for the confounders and the covariates) the potential outcomes are independent. Externally, if this factor is held constant, there is an equivalence between treated and untreated groups; thus, the selection bias is invisible for the purposes of analysis.

These assumptions are essential for making causal inferences using the potential outcome framework. If these assumptions are not met or broken, it can affect the reliability of the causal results obtained from observational data or experiments. Researchers try to use methods or designs in their studies that reduce the chances of breaking these assumptions, helping to find more precise causal connections between treatments and outcomes.

#### 2.1.1. Structural Causal Models

**Structural Causal Models** [3,4,5,6] provide a powerful way to represent and understand the underlying data. By modeling the causal-effect relationship between the variables as a set of clearly structural equations or causal graphs, the SCMs enable us to catch and analyze the causal relationships in this way that is beyond the basic statistical associations. In a SCM, these causal connections are represented through functions, where the input variables (causes) give rise to specific output values (effects). These models are particularly valuable in scenarios where controlled experiments are infected, allowing one to extract causal effects from observational data. The causal relationship through an SCM provides an outline to represent the relationship: a causal graph, which visually represents the causal relationships, and structural equations, which mathematically express these relationships. SCM observation helps to identify the causal mechanisms from data, which is important for making informed decisions in areas such as health and economics.

**Causal graphs** [7] are a type of Bayesian network, the edges indicating the causal effects, which follow the established rules of conditional independence. The graph consists of variable nodes, including treatment and outcomes (observed or uncontrolled), and the directed edges are included, showing the causal relationship between the variables, such as that X → Y represents that X causally affects Y.

**Directed Acyclic Graphs (DAGs)** are used in structural causal models (SCMS), so that causal structures can be represented. They have been designed so that no path in the graph circle is back where it began.

The cause graph in a structural causal model (SCM) represents the causal relationship between variables within a dataset. It acts as a visual guide and reflects the presence of direct causal links and the lack of direct relationships between variables. These graphs emphasize conditional independence relationships, essential to identifying and understanding the system’s causal structure.

To understand conditional independence, the d-separation (dependency-separation) and the concept of a blocked path in graphical models like Bayesian networks or causal graphs are considered. D-separation helps identify when variables are conditionally independent. There are three main ways a path can be blocked between two variables, A and B:

- **Collider Block:** If a collider (a node where two arrows meet) like C lies on the path, the conditioning on C blocks the path. However, this actually allows information to flow through C (A→C ←B).
- **Chain Block:** In a chain (A→C→D→B), where C is not a collider, conditioning on C (or any variable in the chain) blocks the path, stopping the information flow between A and B.
- **Fork Block:** In a forked path (A←C→D←B) with C as the common parent of A and B, conditioning on C blocks the path between A and B.

**Structural equations:** In combination with a causal graph, they enable the specification of causal effects indicated by the directional edges of the graph. These effects can be quantified using a set of non-parametric structural equations, as demonstrated in the causal graph.

(5) $$x=f_{x}\left(\varepsilon_{x}\right),t=f_{t}\left(x,\varepsilon_{t}\right),y=f_{y}\left(x,t,\varepsilon_{y}\right),$$

where $\varepsilon_{x}$, $\varepsilon_{t}$, $\varepsilon_{y}$ represent the “noise” in the observed variables, which can be thought of as unmeasured variations from external factors or independently acting sources.

The noise terms capture how unobserved variables causally affect the variable on the left side of the equation. It is important to emphasize that in each equation, we assume that the variables on the right side of the equation influence those on the left side, and not the reverse.

**Figure 1:**
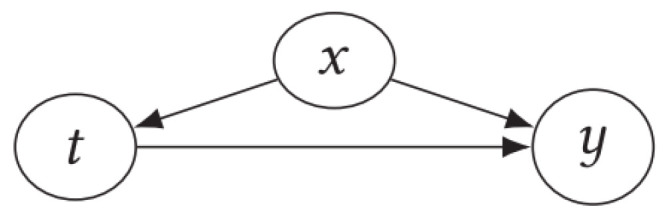
Example of SCM where *x* is confounder, *t* is treatment, and *y* is outcome.

The structural equations offer a quantitative method for representing interventions in a variable within the associated causal graph, as shown in Figure 1.

#### 2.1.2. Potential Outcome Framework

The potential outcome framework [8,9] is widely regarded as the primary approach for studying causal effects, as it focuses on a specific treatment–outcome pair (t,y). This framework addresses a key challenge in causal inference: Only one potential outcome can be observed for each individual in a given scenario. Using this framework, the individual treatment effect (ITE) is defined as the difference between potential outcomes under two different treatment conditions. From this, researchers can derive the average treatment effect (ATE) in a population. Typically, studies involve binary treatments, where t∈{0,1}, with t=1 representing the treatment group and t=0 representing the control group.

The Stable Unit Treatment Value Assumption (SUTVA) is an important assumption in causal inference that simplifies the estimation process by including two key conditions. First, it requires well-defined treatment levels, which means that the treatment given is consistent with the same treatment value. Second, it assumes no interference, ensuring that the outcome of one individual is not influenced by the treatments assigned to other individuals.

Consistency is the second assumption that states that the observed outcomes are independent of the manner in which the treatment is administered. Likewise, unconfoundedness, which is the heart of ignorability, means that potential outcomes are not related to the observed treatments if we consider that all the confounding variables that cause both treatment and outcome to fluctuate at the individual level have been included.

The goal of causal inference within the Rubin causal model is to calculate key quantities such as the Individual Treatment Effect (ITE) for a specific individual. Achieving this requires estimating counterfactual outcomes, which would happen under a different treatment despite the fact that such outcomes cannot be directly observed.

The comparison between structural causal models (SCMs) and the potential outcome framework highlights both their similarities and differences. Logically, the two frameworks are equivalent, meaning that the assumptions made in one can be rephrased in terms of the other. However, the potential outcomes framework focuses on defining causal effects only for the treatment variable and certain specific variables, such as instrumental variables. Although this may seem restrictive, it is a strength because it enables the modeling of specific causal effects without requiring the construction of an entire causal graph. In contrast, SCMs provide the flexibility to study causal effects for any variable, making them better suited for analyzing complex causal relationships involving multiple variables. However, when the goal is to completely estimate the causal effect of a particular treatment, the potential outcomes framework often offers a simpler and more direct method for building estimators.

## 3. Causality Theory

In this section, we introduce causality theory, focusing on two key areas: causal discovery and causal inference. Causal discovery involves identifying causal relationships between variables within datasets. It often addresses challenges such as distinguishing correlation from causation and handling hidden variables. Solutions include using special algorithms and techniques like directed graphs and Granger causality tests. Causal inference is about understanding how treatments or actions affect outcomes. The main challenge is figuring out cause and effect from real-world data, where controlled experiments are not possible. To solve this, methods such as matching scores, using instrumental variables, and comparing differences over time are used. These methods help us to draw better causal conclusions from complex data.

### 3.1. Causal Inference

In the field of causal inference, researchers face many challenges and have developed various techniques to address them. Our focus is on the question, “Would the outcome for this patient have been different if a different medication had been administered?” The resolution of **counterfactual** questions presents a significant challenge for two main reasons. First, we are only able to observe the actual outcome resulting from the administered treatment, rather than the hypothetical outcomes that could have ensued under alternate treatments. Second, in observational studies, treatment assignments are often non-random, which creates differences between the treatment group and the population. This key challenge arises from the inability to observe both counterfactual outcomes and actual outcomes for an individual under different treatment scenarios at the same time.

One of the biggest challenges in causality is handling confounding variables that influence both treatment and outcome. To address these factors, methods such as stratification or regression adjustment are used to separate the actual treatment effect from the effect of external variables. Confounders are specific types of pre-treatment variables, and when calculating the Average Treatment Effect (ATE) by averaging the outcomes of the treated and control groups, the result can be misleading [10]. This is because the ATE may reflect not only the treatment’s effect on the outcome but also the influence of these confounders.

As a result, this can lead to a spurious effect, where the true influence of the treatment is deformed by the presence and effects of confounding variables. The key aspect of causal inference is estimating the average potential outcomes for treated and control groups within a specific population. Focusing on the Average Treatment Effect (ATE), a common approach is to use the average outcomes of the observed variables of the treatment and control groups. However, this method has a major problem due to confounders. The ATE calculated in this way may be a spurious effect because it includes effects caused by these confounders, leading to a misleading representation of the true impact of treatment. This issue highlights the challenge of separating the treatment effect from the influence of confounding variables. Furthermore, confounders impact both the treatment effect and the estimation of the counterfactual outcome. Due to confounder variables, there is a risk of selection bias, which complicates the estimation of counterfactual outcomes. The Table 1 summarizes the solutions proposed in the literature for each problem of causal inference and their limitations.

**Selection bias** occurs when the distribution of the achieved group does not match that of the target group, and thus, there are differences in the distribution of variables between the two groups. This prejudice is often affected by the confectioner and makes it more difficult to estimate counterfeit results. In practice, it involves assessing control results for units in the processed group using data from the observation group, and vice versa, to estimate the results of the treatment for units in the control group on the basis of the observed processed group. The presence of selection bias complicates these estimates. Potential results models can lead to crazy estimates by failing to pay attention to this bias, a problem known as a Covild shift in machine learning. Failing to account for this bias in potential outcome models can lead to inaccurate estimates, a problem known as the covariate shift in machine learning.

In the context of causal inference in real-world scenarios, unobserved confounders are commonly present, which means that the assumption of unconfoundedness is not satisfied. According to structural causal models (SCMs), this situation highlights the inability to block the back-door path by conditioning on observed variables or features alone. The proposed methods generally focus on the idea of using alternative sources of information to account for the presence of unobserved confounders, allowing for more accurate causal inference despite the lack of complete data.

The availability of **large datasets** and the progress in computational methods have introduced both new opportunities and challenges in causal inference. Deep learning and machine learning techniques are increasingly being incorporated into causal inference to manage complex and high-dimensional data, providing more advanced tools to uncover causal relationships [11].

SCM methods may highlight the relationship between variables but may not be able to identify confused effects or build a complete causal graph. In addition, it is important to predict the relationship between these variables for the intervention and evaluation of opposition scenarios. One of the primary challenges at the end of the cause is especially within the structure of potential results, and assessing the treatment effects. In the next part of this section, we will present the methods proposed to solve the challenges with the causes.

**Do-calculus** [3], formulated by Judea Pearl, is a theoretical framework that allows researchers to determine the effects of interventions in causal inference models. It consists of three basic rules that enable the transformation of probability expressions involving interventions, denoted by the ’do-operator’, into standard probability expressions that do not involve interventions. The first rule allows for the addition or removal of observational variables in a causal model when these variables provide no additional information about the outcome given by other variables. The second rule facilitates the exchange between observing and intervening on a variable, under the condition that the variable of interest is independent of the intervention in a modified causal graph. Lastly, the third rule allows the removal of an intervention if it has no additional impact on the outcome given other variables and interventions.

These rules are essential in causal inference because they offer a formal framework for understanding and manipulating causal relationships. Do-calculus is especially valuable when controlled random experiments are not possible. By using these rules, researchers can conclude the cause of the observation data. However, do-Calculus has some limitations, especially because it assumes that variables are independent and distributed by identity (i.i.d.). This performance makes it difficult to produce imaginary distributions at the individual level when using Do-calculus. While do-calculus is able to solve the causal questions, the cause requires prior knowledge of the graph structure, which can limit its practical application.

**Re-weighting** is an effective strategy to mitigate selection bias. This approach assigns different weights to each observation in the data, creating a synthetic population in which the distributions of the treatment and control groups align. Through the assignment of weights, the method aims to correct for selection bias, enabling the dataset to mimic an independent and identically distributed (i.i.d) system. Consequently, less frequent observations are given higher weights, whereas more frequent ones are assigned lower weights. A key concept in re-weighting methods is the balancing score, b(x) [2,10], which is a function of the covariates *x* that ensures the independence of the treatment assignment *W* from the covariates *x*, conditional on b(x). The simplest version of the balancing score is b(x)=x, with the propensity score being a specialized form of the balancing score [12]. This score reflects the probability that an observation will receive treatment (t=1) based on covariates *x*.

Techniques based on the propensity score are some of the most widely used methods in the literature for the correction of selection bias. A widely used method is inverse propensity weighting (IPW), which is sometimes also called inverse probability of treatment weighting (IPTW) [12,13]. For this situation, to improve the efficiency of the estimation capabilities when there are some difficulties in the probability of the event, the so-called doubly robust (Dr) estimator combines the propensity weight with the measurement of the outcome [14]. This method ensures the estimator’s stability as long as at least one model is properly specified, for example, the propensity score or the outcome regression, under the constraint that both are not invalid at the same time. There are also some other methods that enhance the reliability of the score in propensity estimation, such as the covariate balancing propensity score (CBPS) [15]. However, these methods still face difficulties in situations with hidden confounders. Re-weighting methods in statistics are beneficial for reducing bias and enhancing causal inference, but can be complex to implement and sensitive to model accuracy. They use all available data, but may struggle with high variance and unmeasured confounders.

**Matching methods** are a variety of techniques commonly applied in observational studies where random assignment of individuals is not possible but a balance between treated and untreated groups is required. In these approaches, treatment units are matched with control units having the same characteristics/covariates, due to which there exist shared characteristics between them. The central idea of matching is that in a subgroup of units with common covariates, the main difference between the treated and control units is the use of the treatment [10,16].

A well-known method is **propensity score matching** [12], which combines units that have very similar chances of being treated according to their covariates. As a result, the method that gives an answer to this problem is the one which logically connects or causes (successfully) the given information. Secondly, a clustering method of nearest-neighbor matching is selected, which accounts for the dissimilarities in the feature of the treated samples and controls, i.e., the pair of nearest neighbors is selected for each treated unit [17]. In addition, there are matching methods that do not rely on propensity scores, which can address some limitations of score-based techniques [10,18,19]. However, these methods have limitations: They can be limited by the availability of matching partners, potentially leading to a loss of data, and they may not adequately control unobserved confounders. Additionally, the choice of a matching algorithm can significantly impact results, requiring careful consideration and expertise.

**Tree-based methods** utilize decision tree learning, a form of predictive modeling. As a non-parametric, supervised learning approach, decision trees are employed for both classification and regression tasks [20]. The aim is to develop a model that can predict the value of a target variable through simple decision rules extracted from the data. It is specifically effective in identifying heterogeneous treatment effects, which means that the effect of an intervention is distributed differently between subgroups. The Bayesian additive regression tree (BART) model [21] is a development of the classification and regression tasks model, similar to the relationship between random forests and decision trees. This multimodel approach leads to smaller, less complex trees compared to a single tree in CART, functioning as weak classifiers. By employing multiple trees, BART effectively captures a wide range of covariate effects, offering robustness and greater accuracy than single-tree models [10].

Tree-based methods are highly effective at capturing complex, non-linear relationships and detecting heterogeneous treatment effects. However, they risk overfitting complex datasets and can struggle with confounders and sparse data, requiring careful tuning for accurate causal analysis [22,23]. Each of them has its own strengths and weaknesses, and therefore, choosing a suitable technique often relies on the context of the research and the parts of the datasets.

**Table 1:** Summary of solutions to causal inference problems.

Problems	Solution	Advantages	Limitations
Causal question	Do-Calculus [3]	- Efficiency, - Flexibility, - Robustness, - Generalizability	- Need causal graph’s structure, - Assumption of i.i.d, - Selection bias
Selection bias	Re-weighting [2,10]	- Reducing bias, - Enhancing causal inference	- Unmeasured confounders, - Complex to implement and sensitive to model accuracy, - Struggle with high variance
Selection bias	Matching methods, propensity score matching [12]	- Reduces selection bias, - Improves the validity of causal inferences	- Do not control unobserved confounders, - Choice of matching algorithm
Treatment effects	Tree-based methods [20]	- Handling complex, non-linear relationships, - Identifying varied treatment effects	- Risk overfitting with complex datasets, - Struggle with confounders and sparse data, - Requiring careful tuning for accurate causal analysis
Confounding variables	Regression adjustment	- Handle multiple confounders, including continuous variables	- Correct model specification

### 3.2. Causal Discovery

Causal discovery refers to the process of uncovering cause-and-effect relationships between variables within a dataset. It goes beyond identifying correlations by distinguishing true causal links, using statistical and computational techniques. This approach is essential in various fields to understand how changes in one variable directly impact another. The main challenge in causal discovery lies in determining causation from observational data. Since correlation does not imply causation, variables may exhibit complex and hidden interrelations. The presence of **confounding variables** that affect both the cause and the effect can further complicate the analysis and lead to inaccurate conclusions. Additionally, the lack of experimental control in observational studies makes it difficult to isolate variables and definitively establish causal relationships. The main problem is to select an appropriate causal graph that explains the observed data [24]. This graph represents the cause-and-effect relationships among the variables, providing critical insights. Methods for addressing causal discovery are generally categorized into three main approaches [11]: constraint-based methods, score-based methods, and non-Gaussian methods. The Table 2 summarizes the solutions proposed in the literature for each problem of causal discovery and their limitations.

**Constraint-based methods** involve acquiring a set of causal graphs that satisfy the conditional independence implied within the dataset [25]. Statistical tests play a crucial role in confirming whether a proposed graph satisfies this independence, using the faithfulness assumption [26]. Faithfulness implies that the statistical connections observed between variables in the data do not contradict the independence specified by any causal graph producing that data. Constraint-based methods are based on the algorithm of inductive causation [27,28], which infers potential causal relationships within a given set of data. This algorithm employs statistical tests to identify dependencies and independencies among variables, helping to construct a causal graph that represents these relationships. The IC algorithm is particularly notable for its ability not just to suggest direct causal links but also to indicate when causal relationships cannot be determined from the data alone. The steps of the IC algorithm are difficult to implement. Various algorithms build on IC’s concepts to offer practical methods with no hidden confounders in the model. The SGS algorithm [29], however, has exponential complexity in the worst case. A more popular alternative is the Peters and Clark (PC) algorithm [26], which operates in polynomial time. To enhance PC’s efficiency and stability, several improvements have been made. These include reducing the number of conditional independence tests and easing assumptions about node order, as seen in variations like PC-stable [30], conservative-PC [31], PC-select (originally named PC-simple) [32], and a min–max heuristic-based PC method (MMHC) [33]. The IC* algorithm proposed to adapt by including hidden confounders [34] alters its third step to accommodate the impacts of confounding factors. This modification enables it to recover the Markov-equivalent class effectively. The fast causal inference (FCI) algorithm [26] enables the implementation of IC in the presence of confounders, while the real fast causal inference (RFCI) algorithm [35] speeds up its execution. The more recent iterative causal discovery (ICD) method [36] reduces the number of conditional tests by using an iterative approach. As the graph becomes sparser with edge removal, this method maintains speed even with a large set of conditional variables.

**Score-based methods** involve evaluating potential causal graphs for a set of variables by calculating a specific score to determine their fit. Given the huge number of possible graphs, the main challenge of these methods lies in efficiently reducing the search space [25]. One widely used scoring function in these approaches is the Bayesian information criterion (BIC). For instance, the GES (greedy equivalence search) algorithm adapts its scoring functions based on the type of data being analyzed: It uses the BIC score for continuous data, a Bayesian Dirichlet uniform score based on likelihood equivalence for discrete data, and the Conditional Gaussian score for datasets with both continuous and discrete variables. Several other approaches, such as fast GES and the K2 algorithm, also employ score-based methods for causal inference.

These methods commonly employ the Bayesian information criterion (BIC) score to evaluate potential causal graphs. Specifically, the GES algorithm [37] adjusts its scoring function based on the type of data it analyzes: using the BIC score for continuous variables, a score based on the likelihood-equivalence Bayesian Dirichlet uniform distribution for discrete variables, and the conditional Gaussian score for mixed data, which comprises both continuous and discrete variables. The K2 algorithm [38], an earlier method, adopts a greedy strategy to select parent nodes by their probability contributions, although it does not completely account for conditional independence due to its assumptions on data distribution. The greedy equivalence search method [37,39] iteratively builds a causal graph by first adding and then removing edges to improve the score, assuming there are no hidden confounders. This process has been further refined by the greedy interventional equivalence search (GIES) [40] and the fast greedy equivalence search (fGES) [41], which introduce interventions and improve scalability, respectively. Additionally, the greedy fast causal inference (GFCI) method [42] extends these approaches to include hidden confounders by merging GES with the FCI algorithm, offering a more comprehensive solution for causal inference.

**Non-Gaussian methods**. Non-Gaussian methods are essential for analyzing data that do not follow normal distributions, common in many real-world scenarios. These methods, unlike Gaussian-based ones, exploit asymmetries in data distributions to infer causal relationships. These methods are particularly useful for identifying causal relationships in complex and nonlinear datasets. Key approaches include independent component analysis (ICA) [43], which separates non-Gaussian components in multivariate data, and additive noise models (ANMs) [44,45], which use non-Gaussian noise to identify causal directions. These methods are valuable in diverse non-linear datasets. Moreover, these methods operate under the assumption that the data are free of confounders.

The linear non-Gaussian acyclic model (LiNGAM) [46] relies on several key assumptions: linearity, non-Gaussian continuous error variables (with at most one exception), acyclicity, and the absence of hidden common confounders. These assumptions allow LiNGAM to effectively model and analyze causal relationships in datasets where variables follow non-Gaussian distributions. The ICA-LiNGAM algorithm, developed based on independent component analysis (ICA), is specifically designed to uncover causal relationships within the LiNGAM model framework [46]. This approach leverages ICA’s strengths to better learn and interpret causal links within the LiNGAM model, enhancing its capability to analyze complex datasets with non-Gaussian distributions. Algorithms like ICA-LiNGAM are adaptable for time-series analysis. An example is the auto-regressive LiNGAM, designed to learn causal relations from time series data [47].

## 4. Causality for Machine Learning

In machine learning, the integration of causality goes beyond mere pattern recognition, focusing on understanding cause and effect. Traditional machine learning models are adept at finding correlations, but often confuse these with causation. This can lead to unreliable predictions under new conditions. Causal learning addresses this by embedding causal inference principles, allowing models to anticipate the impact of changes or interventions. This shift not only improves the reliability and adaptability of the model, but is also crucial for interpretability and ethical decision-making in critical areas like healthcare and policy-making, where causal understanding is essential. Traditional methods for identifying causality often face challenges when working with high-dimensional variables. Although machine learning excels at uncovering patterns in large datasets in various tasks, it tends to struggle when trying to apply these patterns beyond the original range of the data. It also faces challenges in understanding cause and effect and can be misled by confounding variables [48,49,50,51]. Recently, efforts are being made to blend machine learning with causal reasoning. However, creating neural models that effectively reason about causes is still in early development, and building these on a large scale is an ongoing challenge in the field.

Integrating causality for machine learning models poses several complex challenges, particularly in terms of generalization, modularity, interventions, and counterfactuals. In machine learning, **the generalization** challenge arises from assuming data are independent and identically distributed, which can make models unreliable in different settings [48,49,50,51]. For example, a model might work well with one type of image, but not with another. This leads to issues such as bias and susceptibility to misleading data. To combat this, there is a shift towards using larger models and more data. However, causal models offer a different approach. They focus on the underlying causal structures of tasks, making them less dependent on specific data distributions and more robust in situations with limited data. Modularity in deep learning (DL) relates to the design of more interpretable and less computationally intensive systems. Current DL models are often complex “black boxes”, making them hard to interpret and compute intensive. Modular architectures in deep learning are advocated for their ability to improve learning efficiency and applicability across various tasks [52,53,54]. The concept of modularity, supported by principles such as the independent causal mechanism (ICM) [55,56] and the sparse mechanism shift (SMS) hypothesis [56], suggests that causal mechanisms are autonomous and do not interact with each other. This means that changes in the system should only affect local parts of a causal model, avoiding unnecessary dense computations that can lead to spurious correlations, interventions, and counterfactuals. Current machine learning (ML) models struggle with answering “what if” queries, which are essential for causal reasoning at advanced levels. Although reinforcement learning (RL) can approach some of these queries, the integration of causal reasoning could further enhance the ability of RL models to imagine and respond to hypothetical scenarios [56,57].

**The Neural Causal Model** (NCM) [5] is a specialized type of structural causal model (SCM) that integrates neural networks for causal inference. It addresses the limitations of neural networks in predicting intervention effects from observational data alone. NCMs are designed with structural constraints necessary for causal reasoning. They are a specific category of structural causal models (SCMs) characterized by certain constraints. They are used for tasks such as causal identification and estimation, leveraging neural network tools for these purposes. NCMs also help to evaluate counterfactual scenarios, which are important for applications in AI such as fairness assessment and responsibility determination. The causal links between variables in these models are represented using a specific network called multi-layer perceptrons (MLPs) [58], which have adjustable parameters. Despite these limitations, NCMs are still versatile and can handle various types of causal questions, including observations, interventions, and counterfactuals [5]. However, it is important to note that using MLPs for causal inference can be complex in real-world applications.

**The Tractable Neural Causal Model** (TNCM) [58] is an enhancement of the standard neural causal model (NCM). In TNCM, the multi-layer perceptrons (MLPs) used in NCMs are replaced with sum-product networks (SPNs) [59,60]. An SPN is a type of directed acyclic graph (DAG) that starts from a root and has leaf nodes that represent probability distributions over causal variables. Unlike typical DAG conventions, the root node in an SPN indicates the output, which, in this context, is the probability of a variable *y* given another variable *x*. The intermediate nodes in an SPN are not mere data points, but perform either sum or product operations, each parameterized in its way. This structure allows the TNCM to effectively handle complex probabilistic relationships in causal modeling.

The GNN-SCM [60] is a novel neural causal model that uses graph neural networks (GNNs) instead of the commonly used multi-layer perceptrons (MLPs) for causal inference. In this model, the structure of the causal graph is the same as the input graph in the GNN; the variables are expressed as nodes in the graph. The parents of a node in the directed acyclic graph (DAG) correspond to its neighboring nodes in the GNN. The probability of a node given its parents returns the output of the GNN. Interventions in a GNN-SCM are defined by modifying the graph structure: specifically, by removing the parents of the intervened node from the set of its neighbors. This approach leverages the natural fit of GNNs to represent graph structures, which aligns well with the requirements of structural causal models.

The flexibility of comprehensive causal machine learning (CCML) methods, presented in [61], makes them valuable for estimating causal effects at different levels of granularity. CCML methods, including dml, grf, and mcf, effectively estimate causal effects at the granular levels. mcf stands out for its robustness to selection bias and internal consistency. Causal reinforcement learning integrates causality into reinforcement learning to enhance data efficiency and interpretability [62]. This survey categorizes causal reinforcement learning methods into those with known and unknown causal structures, analyzing their applications across Markov decision processes, partially observable Markov decision processes, multi-armed bandits, imitation learning, and dynamic treatment regimes. It reviews evaluation metrics, open-source resources, and emerging applications. Some researchers use causality to analyze fairness in machine learning, with the objective of uncovering hidden biases and ensuring fair decision making. The paper [63] explores how the disparities in AI decisions stem from underlying causal mechanisms rather than just observed data. It introduces the Fairness Map, which organizes fairness criteria, and the Fairness Cookbook, which provides guidelines to assess unfair treatment, such as disparate impact and disparate treatment. These causal approaches help researchers move beyond correlation-based fairness measures, making machine learning systems more interpretable, accountable, and equitable.

The causal intervention graph neural network (CIGNN) [64] provides a novel approach to fault diagnosis in complex industrial systems by integrating causal theory with graph neural networks. Traditional graph-based models struggle with confounding effects caused by irrelevant sensor signals, leading to inaccurate predictions. To address this, CIGNN constructs structural attribute graphs using an attention mechanism, capturing the relationships between sensor signals and faults. Then, it applies causal interventions using an instrumental variable to separate true causal features from confounding influences. Experimental results in industrial datasets confirm that CIGNN significantly improves fault diagnosis accuracy, interpretability, and robustness.

## 5. Causal-Based Features Selection Methods

In the realm of machine learning and high-dimensional data analysis [65,66,67,68], feature selection is a crucial process. It involves selecting a robust subset of features from larger features predictors to construct effective models for a specific target or class variable. This is more important now than ever because we often deal with complex and high-dimensionality datasets in many areas. Without feature selection, most machine learning methods struggle with datasets of very high dimensionality. Over the last 20 years, there has been a lot of progress in feature selection. It has helped make learning from data less computer-intensive and enhances model generalization and can better predict new data [67]. Feature selection is now more crucial than ever in the field of data science. This is because datasets with a large number of dimensions are now common in various applications [69]. With an increase in data complexity, choosing the right features becomes a key to effective data analysis and model building.

A traditional feature selection method aims to identify a subset of important features based on their associations with the target variable. Generally, these associations are correlations that only show how features and the target variable appear together, not causal relationships [66]. Recent research suggests that the identification of causal features can offer significant advantages in feature selection for classification purposes, enhancing the effectiveness of the model [70,71]. We address a critical challenge in the field of machine learning using causal-based feature selection: the identification and selection of features that not only contribute to the predictive accuracy of models, but also adhere to robust causal principles. Traditional feature selection methods often prioritize correlation over causation, leading to models that could perform well in training data but fail to generalize to new or unseen data due to spurious correlations or overlooked confounding factors [72]. The core problem in this domain is the development of a feature selection methodology that integrates causal inference and discovery techniques to ensure that the selected features have a genuine and robust causal relationship with the target variable [70,73]. This approach aims to enhance the generalizability, interpretability, and reliability of the models. The research explores innovative causal inference methods, evaluates their effectiveness in various machine learning scenarios, and seeks to establish a framework that can be applied effectively in different domains and data types.

Feature selection in machine learning is divided into various categories, including filter, wrapper, embedded, and hybrid approaches. Each category signifies a distinct strategy for integrating feature selection processes with learning algorithms. **Filter approaches** utilize statistical tests and operate independently of predictive models. In contrast, **wrapper methods** evaluate combinations of features through a predictive model, embedded approaches integrate the feature selection process directly into the model training phase, and **hybrid methods** combine aspects of the other three types for a more integrated approach [74]. Filter methods are fast and unbiased towards specific models, making them increasingly popular for high-dimensional data. They classify features as highly relevant, weakly relevant, or irrelevant to the outcome variable [75].

**Wrapper methods** rely on a learning algorithm to identify features based on their impact on the algorithm’s performance. These methods follow a two-step process: selecting a subset of features and evaluating their performance using the learning algorithm. This process is repeated until optimal performance is achieved or an accepted number of features is selected. However, a major limitation of wrapper methods is their inefficiency with large datasets, as the number of possible feature combinations can be overwhelming. Although search strategies such as sequential search [66] or best-first search [76] can help narrow the search space, they do not fully address the scalability problem. Consequently, wrapper methods are rarely applied to high-dimensional data.

**Filter methods** for feature selection work independently of learning algorithms, using data characteristics to determine the importance of features. They are generally more efficient than wrapper methods. The process involves two steps: ranking features based on certain criteria, and then filtering out the less important ones. The ranking can be univariate (each feature is ranked alone) or multivariate (features are ranked together). However, since these methods do not use a specific learning algorithm for selection, the chosen features might not be the best fit for the intended algorithm. Various criteria are used to decide whether a feature should be kept during each iteration of feature selection. These include measures of association, divergence, and how well machine learning (ML) models perform with the feature. The variance metric assesses feature divergence but does not account for the relationship between input and output features [77]. The Pearson correlation coefficient (PCC) chooses features that are linearly related to the target [78]. The maximal information coefficient (MIC) identifies non-linear relationships between two variables, but requires more data and might underestimate the total association [79]. Feature selection using these criteria is independent of ML models and falls under filtering methods.

**Embedded methods** mix the best parts of filter and wrapper methods by including feature selection as part of the learning process. This strategy brings together the best of both worlds: It considers how features interact with the learning algorithm and avoids the time-consuming process of repeatedly testing different sets of features, which is a drawback of wrapper methods. The most popular embedded methods use something called regularization models. These models seek to achieve a good fit for the data while also keeping the sizes of the feature coefficients either small or reducing some to zero. This process effectively selects important features and provides both the optimized model and the selected features in the final output.

It is important to recognize that certain studies categorize feature selection techniques into four groups from the perspective of the selection method, adding hybrid feature selection methods as a distinct category (as noted in [80,81]). Hybrid methods are essentially a blend of several feature selection techniques, such as wrapper, filter, and embedded techniques. Their primary aim is to address the issues of instability and sensitivity to data perturbations that are common in many feature selection algorithms. For example, in the case of datasets that are high-dimensional but small in size, even minor changes to the training data can lead to vastly different feature selection outcomes. Hybrid methods enhance the stability and reliability of feature selection by combining multiple subsets of features obtained from different selection techniques, thereby improving the trustworthiness of the selected features.

Regarding the challenge of selecting features, methods such as embedding and wrapper involve a machine learning training process that incurs significant computational expenses. Their effectiveness is closely related to the machine learning models chosen. However, filter methods, including those based on variance, the Pearson correlation coefficient (PCC), and the maximal information coefficient (MIC), operate independently of machine learning models. They work by selecting a feature subset through a pre-set manual threshold. Common thresholds involve a predetermined number of features or a specific variance, PCC, or MIC. However, setting a meaningful and theoretically grounded stopping threshold is often a complex task with limited interpretability [82].

In recent years, feature selection methods based on causality principles have been developed. These methods utilize Bayesian network (BN) and Markov boundary (MB) and are applied in both machine learning and causal discovery to identify features with potential causal relationships [70,83,84]. A Bayesian network is represented as a directed acyclic graph (DAG), where nodes correspond to variables, and edges depict dependencies between them. When an edge from X to Y is interpreted as X causing Y, the BN becomes a causal BN, representing causal relationships. A distinctive aspect of a BN is its Markov blanket (MB), which includes a variable’s parents (causes), children (effects), and spouses (co-parents of a variable’s children). This property holds under the assumption of faithfulness [85].

The feature selection algorithms based on causality proposed in the literature are divided into two categories: constraint-based and score-based methods. These approaches provide a unique and complementary strategy for improving feature selection, particularly to achieve explainable and robust machine learning models [85]. Forward-backward feature selection operates in two phases: a forward phase that adds features and a backward phase that removes them, continuing until specific criteria are met. The traditional approach performs these phases sequentially, while the interleaving approach alternates between adding and removing features [86].

**Constraint-based methods** can be grouped into five main categories [85]: simultaneous Markov blanket (MB) learning, divide-and-conquer MB learning, MB learning that combines interleaved PC and spouse discovery, MB learning under relaxed conditions, and specialized MB learning approaches. **Simultaneous MB** learning focuses on identifying the parents, children, and spouses of a target variable using a combined forward–backward approach, without explicitly separating parents and children from spouses during the process. The key algorithms in this category include GSMB [87] and IAMB [88] and its variants. GSMB was the first algorithm to learn an MB without requiring a complete Bayesian network. Inter-IAMB [86] alternates between the phases of IAMB, while FBED [83] and PFBP [89] are more advanced versions of IAMB. Simultaneous MB learning algorithms are computationally efficient as they minimize the number of tests performed; however, they require more data for each test, making them less effective for small sample sizes. These algorithms perform best when the Markov blanket (MB) of the target variable is relatively small [85].

**The divide-and-conquer MB learning approach** reduces data requirements by dividing the learning process into two steps: identifying the parents and children (PC) of a target variable and then its spouses (SP). It is more data-efficient than simultaneous MB learning as it uses smaller subsets for tests, but it is less time-efficient, especially with larger feature sets. Key algorithms include MMMB, HITON-MB, and PCMB, among others [85]. While this method needs fewer samples, it becomes computationally demanding with more features.

**MB learning with interleaved PC and spouse learning** extends the divide-and-conquer approach by alternating between learning PC (parents and children) and identifying spouses. When a candidate for PC of C is found, it immediately activates the spouse learning phase. Notable algorithms include BAMB and EEMB, with BAMB handling both PC and spouse candidate sets simultaneously, and EEMB splitting these into separate learning and pruning steps. This interleaving aims to balance data and time efficiency by keeping candidate sets small. However, incorrectly included PCs can lead to large spouse sets, affecting the effectiveness of both BAMB and EEMB [85].

**MB learning with relaxed assumptions** modifies traditional algorithms that rely on faithfulness and causal sufficiency, both often violated in practice. When faithfulness is violated, multiple MBs may exist for a target variable. To address this, algorithms such as KIAMB, TIE*, SGAI, LCMB, and WLCMB identify multiple MBs without relying on faithfulness [85]. KIAMB, the first to try this, can not guarantee finding all MBs and needs multiple runs. TIE* finds all MBs but is computationally intensive. SGAI is more efficient but might miss some MBs. WLCMB, similar to KIAMB, shares its limitations. When causal sufficiency is violated, traditional MB learning algorithms might not accurately reflect true causal relationships.

**MB learning with special purposes** includes algorithms designed for unique scenarios. MIMB identifies an MB from multiple datasets, while MCFS focuses on stable prediction amid distribution shifts. MIAMB and MKIAMB learn MBs for multiple class variables. BASSUM and Semi-IAMB are tailored for weakly supervised MB learning. These approaches demonstrate how causal feature properties aid in semi-supervised learning and feature selection, especially when distribution shifts. The application of machine learning methods along with causal discovery is becoming more appealing for the subjects in addition to feature selection [85].

The current **score-based MB learning algorithms**, which were inspired by constraint-based methods, discover a directed acyclic graph (DAG) around a target variable and recognize its MB. There are three categories of these algorithms: divide-and-conquer MB learning, simultaneous MB learning, and MB learning with relaxed assumptions.

**The SLL algorithm**, according to the paper [90], is a score-based variant of the divide-and-conquer MB learning method. It adopts a Bayesian network (BN) structural learning methodology for learning parents, children (PC), and spouses separately. SLL applies symmetric checks with AND and OR rules to eliminate false positives from PC and spouse sets, respectively. However, this makes SLL computationally demanding, especially for large MBs. To enhance efficiency, the S2TMB algorithm [91], a score-based variant of STMB, was introduced. It identifies spouses differently and uses identified spouses and PC for false positive removal, bypassing symmetric checks. S2TMB+ further refines this for improved computational efficiency. The algorithms DMB and RPDMB [92], unlike SLL and S2TMB, learn parents and children (PC) and spouses of a target variable C at the same time, not in separate steps. The fGES-MB algorithm is an adaptation of the fast GES (fGES) algorithm, incorporating optimizations like score caching and parallelization to speed up the process and handle high-dimensional data. Given fGES’s efficiency, fGES-MB is also capable of dealing with high-dimensional datasets.

In the case of violation of the faithfulness assumption, BSS-MB was introduced as a variant of KIAMB, a score-based method for learning multiple Markov blankets (MBs). In cases where causal sufficiency is violated, LMB-CSEM was the first score-based method capable of learning MBs with latent variables in a directed acyclic graph (DAG). However, BSS-MB sometimes fails to identify all the MBs it should and provides no significant improvement over KIAMB or TIE* in terms of computational efficiency or accuracy. LMB-CSEM utilizes the EM algorithm to handle missing values related to latent variables, but this approach becomes computationally expensive when applied to large datasets [85].

In conclusion, using score criteria for Markov blanket learning when the faithfulness or causal completeness assumptions are violated remains a challenge. Current methods, which are still grounded in the constraint-based MB learning framework, do not offer significant advancements over traditional constraint-based approaches, putting less emphasis on causality-driven feature selection studies [85].

In the paper [93], the authors introduce the FSNT method for feature selection in high-dimensional data, enhancing accuracy in regression and classification models. FSNT, based on NOTEARS causal discovery, optimizes feature selection by identifying crucial causal relationships. Its performance is tested using various regression and classification algorithms in multiple datasets. The results show a substantial improvement in model prediction accuracy, with FSNT outperforming other feature selection methods, especially in reducing regression errors and increasing classification precision.

In the paper [94], a new algorithm is introduced to identify direct causal factors from numerous explanatory variables, addressing challenges with scalability, nonlinear relationships, and cyclic data. Using a one-vs.-the-rest feature selection approach and inspired by debiased machine learning methods, it effectively handles observational data, including nonlinear contexts with cycles. Efficient even for large datasets, this algorithm shows significant improvements over traditional methods in causal inference.

Feature selection is essential for the prediction of disease risk in machine learning, addressing the curse of dimensionality in genomic data [95]. The review [95] highlights filter, wrapper, embedded, and hybrid methods, with hybrid approaches emerging as best practice by combining efficiency and accuracy. Selecting informative single nucleotide polymorphisms (SNPs) improves prediction, reduces computational costs, and enhances disease understanding. Integrating biological knowledge into feature selection further improves interpretability, making it crucial for precision medicine.

The attention-based gated recurrent unit with Granger causality (AttGRU-GC) framework [96] enhances the prediction of industrial process quality by selecting the most relevant input variables based on causal relationships. By integrating deep learning with Granger causality, the model effectively captures non-linear and dynamic dependencies between process variables and quality outcomes. The attention mechanism further improves prediction accuracy by focusing on significant time-lag dependencies. The experimental results confirm that AttGRU-GC outperforms state-of-the-art methods in variable selection and predictive modeling.

The study [97] introduces a two-stage feature selection method to enhance water quality prediction by identifying the most relevant variables while reducing computational complexity. Combining filter and wrapper methods, this approach ensures the optimal selection of a subset of feature to predict effluent total nitrogen (E-TN) in wastewater treatment plants. The golden jackal optimization (GJO) algorithm further refines feature selection, improving model accuracy. By integrating selected features into a CNN-LSTM-TCN hybrid model, the system effectively captures non-linear relationships in time series data. The results show that optimized feature selection significantly improves prediction performance, outperforming traditional methods.

The survey [98] explores different causal feature selection methods used in responsible machine learning. It introduces a new taxonomy that categorizes methods based on their application to key ML challenges, including interpretability, fairness, adversarial robustness, and domain generalization. The study highlights that current fairness-focused methods rely on causal graphs, which can be limiting in real-world settings, and suggests developing new approaches that work without prior causal knowledge. For adversarial robustness, causal feature selection is used to explain attacks, but future research should focus on making models more resistant to them. In domain generalization, existing methods identify cause–effect pairs or Markov blankets, but incorporating multimodal data and temporal dynamics could improve performance. Advancing these methods will help create more transparent, ethical, and robust machine learning models.

**Causality-Driven Feature Selection in Sensor Data Applications**. Causality-driven feature selection addresses the limitations of correlation-based methods by identifying true causal relationships, improving model reliability and interpretability. This approach is crucial in sensor-based applications, where data are often noisy and interdependent. The following paper analyzes recent advances in this field, highlighting their impact across various domains: In it, [99], the authors address the limitations of traditional correlation-based feature selection methods for multi-dimensional sensor data by introducing Max-JDI (joint directed information maximization), a causality-driven metric capturing both instantaneous and temporal dependencies. To solve the computational challenges of high-dimensional data, the authors propose an efficient greedy algorithm. Evaluated on a real-world power distribution dataset, their method improves detection accuracy by 5% and reduces computation time from weeks to less than a minute. This approach enhances reliability and scalability in causal feature selection, with significant applications in smart energy systems.

The paper [100] proposes a causal feature selection method to calibrate low-cost sensors (LCS) to address the limitations of traditional machine learning methods based on statistical correlations. Using convergent cross-mapping, the approach selects features with genuine causal relationships, improving accuracy and robustness in dynamic environments. Applied to the calibration of the OPC-N3 sensor for PM_1_ and PM_2.5_, it reduced the mean squared error by 43.2% and 33.2%, respectively, outperforming SHAP-based selection. This method enhances sensor calibration by ensuring interpretability, generalizability, and adherence to Occam’s razor, with broader applications in environmental monitoring.

The paper [82] presents a new automatic feature selection method for industrial soft sensing, focusing on key performance indicators (KPIs). This method, inspired by the post-nonlinear causal model and integrated with information theory, identifies features with significant causal effects on KPIs, using the Shannon entropy. Then, it uses an AdaBoost ensemble strategy to develop soft sensors. Tested in two industrial settings, this approach shows promise for enhancing data-driven soft sensors in various industrial processes.

The paper [101] presents a distributed deep Bayesian LSTM prediction framework to improve trend prediction in multi-sensor systems while addressing data noise and redundancy. The causality entropy method is introduced for feature selection, where the series causality coefficient (SCC) identifies the most relevant sensor measurements, reducing unnecessary data input and computational load. To handle sensor noise, the model employs a Bayesian LSTM, using weight sampling to improve prediction stability. A multilayer perceptron (MLP) fusion layer integrates outputs from sub-predictors, further enhancing accuracy. The results were tested with Beijing meteorological data, which confirmed that this approach significantly improves prediction performance, demonstrating its effectiveness in handling large and noisy sensor datasets.

The study [102] emphasizes the role of feature selection in intelligent fault diagnosis (IFD) of manufacturing processes using sensor data. The proposed Extra Tree (ET) classifier, combined with SHapley Additive exPlanations (SHAP), selects the most relevant sensor features, filtering out noise and redundant measurements. Using essential sensor data, the model improves fault classification accuracy while reducing computational complexity. Unlike deep learning models, which require extensive preprocessing, the ET-based approach processes sensor inputs efficiently, achieving a greater accuracy of 99% with lower resource consumption. Future research could explore automated sensor feature selection and Bayesian optimization to further enhance fault detection and root cause analysis.

The paper [103] proposes causal network-based feature extraction methods to improve fault detection and diagnosis (FDD) in rail transport systems using sensor data. With modern railway systems relying on high-dimensional sensor datasets, traditional correlation-based dimension reduction methods struggle with interpretability and robustness. To address this, the study constructs causal networks from railway sensor data, using causal strength matrices to identify fault-relevant features. These methods effectively reduce redundant sensor signals while preserving key causal information and improving fault detection accuracy. Tested on two public datasets and a real high-speed train (HST) braking system dataset, the proposed approach outperforms classical PCA, KPCA, and ICA in stability and effectiveness. Future research will integrate expert knowledge with sensor-based causal networks and explore multifault detection for enhanced fault diagnosis in railway systems.

The JITL-MBN method [104] introduces a real-time causality representation learning approach for sensor fault diagnosis in high-speed train traction drive systems (TDS). The method leverages sensor data from multiple TDS modules, including rectifiers, inverters, and DC links, to detect faults that propagate between different components. By integrating just-in-time learning (JITL) and modular Bayesian networks (MBN), the model efficiently analyzes sensor relationships, identifying causal dependencies between faults and sensor readings. MBN structures the causal connections among sensor signals, improving interpretability and reducing computational complexity. JITL enhances real-time adaptability by using K-means clustering to select relevant sensor data, reducing retraining time. The experimental results demonstrate that JITL-MBN outperforms traditional classification methods, achieving superior accuracy and speed for sensor fault diagnosis.

## 6. Conclusions

In this review, we provided an in-depth overview of the challenges and solutions within the field of causality, with a particular focus on causal inference and discovery. We explored how causality is integrated into machine learning models, emphasizing how causal principles can enhance predictive accuracy. In addition, we analyzed causal-based methods for feature selection in sensor data, focusing on their role in improving the effectiveness of machine learning applications. By addressing these challenges and proposed solutions, this paper aims to provide a comprehensive understanding of how causality can be leveraged to enhance the performance and reliability of machine learning models. In conclusion, while the convergence of causality and machine learning presents significant opportunities to advance the field, it also introduces unresolved challenges that require further investigation and development.

Open challenges at the intersections of causality, machine learning, and causal-based feature selection are at the forefront of current research efforts. These challenges are critical to advance the field and unlock more sophisticated applications. Some key open challenges are defined as follows:

- Scalability: developing methods that effectively handle high-dimensional datasets.
- Deep Learning Integration: merging causal inference with deep learning for improved interpretability and performance.
- Feature Selection: enhancing causal-based feature selection methods for complex models.
- Generalization: ensuring causal models generalize well across different domains.

## Figures and Tables

**Table 2:** Causal discovery methods and their characteristics.

Problems	Solution	Methods	Advantages	Limitations
Causal graph	Constraint- based methods	IC algorithm	- Construct a causal graph Flexibility with Data Types	- Difficulty in Handling Large-Scale Data Difficult to implement no hidden confounders
		PC algorithm	- Reducing the number of conditional independence tests - Easing assumptions about node order	- No unmeasured confounders
		IC* algorithm	- Handling hidden confounders	- Complexity - Unobserved variables
		FCI algorithm	- Handling of Latent Confounders and Selection Bias	- Computational Complexity - Harder to apply in the case of confounders and bias
	Score-based methods	K2 procedure	- Suitable for discrete variables - Interpretable and less complex structures	- Limited to discrete data - Not perform as well as GES or Fast GES
		GES	- Flexible algorithm that can be used for both discrete and continuous data - Discovering both the direction and existence of edges between nodes in a Bayesian network	- No hidden confounders
		fGES	- Flexible algorithm that can be used for both discrete and continuous data - Discovering both the direction and existence of edges between nodes in a Bayesian network - Fast running	- Sensitive to complex or noisy data
	Non-Gaussian methods	ICA	- Independence Assumption - Capturing both linear and nonlinear causal relationships in data	- Computational - Complexity for large datasets or complex nonlinear relationships
		ANMs	- Well suited for large datasets - Requires causal ordering	- Linearity assumption - Gaussian noise assumption
		LiNGAM	- Computationally efficient for large datasets - Interpretable results	- Linearity assumption - Gaussian noise assumption
